# News and Notes

**Published:** 1908-12

**Authors:** 


### NEWS AND NOTES

---

Dr. and Mrs. Dunbar Roy have returned from their visit to New York.

---

Dr. Ed Crawford and Miss Carol Gray were married at College Park on October 25, '08.

Dr. T. V. Hubbard had his arm broken and was otherwise painfully bruised in an automobile accident recently.

---

Jonathan Hutchinson, Sen., T. R. S., F. R. C. S., was recently conferred to honor of Knight by King Edward.

---

Cincinnati University is to have a new building for the College of Medical Sciences to cost $275,000, besides $25,000 for equipments.

---

Cooper Medical College, San Francisco, began its career as an integral part of Leland Stanford, Jr., University, November 3. It will henceforth be known as the school of medicine of the university.

---

The marriage of Dr. J. Edgar Paulin and Miss Edna Frederic of Marshallville, Ga., will be an event of wide interest.

Though Dr. Paulin has made his home in Atlanta only recently, he is proving himself as welcome professionally as his charming bride will be socially.

---

The doctors of the Third Congressional District held their fourth annual meeting at Montezuma, Ga., on Wednesday, Nov. 18, about twenty-five physicians were in attendance and several interesting papers were read. Americus was chosen as their next meeting place.

---

The Southern Medical Association holding their annual convention here were beautifully entertained by the physicians of Atlanta at a reception at the Piedmont Driving Club.

The guests were received by Dr. Willis Westmoreland, chairman of the reception committee, and a large number of Atlanta's most prominent men and women.

John A. Taff, an osteopath of Boston, formerly of Louisville, Ky., charged with practicing medicine without being registered as a physician, was sentenced to imprisonment for three months in the House of Correction.

---

At the recent celebration of "Academic Day" of the University of Maryland, it was decided to erect a tablet in memory of the late Major James Carroll, of the Army Medical Corps, who received his diploma from this University.

---

Students of the senior class of the Medical Department of the University of Buffalo, presented Dr. Roswell Park with a silver service in honor of his completion of a quarter of a century as professor of surgery in the institution.

---

Regarding the North Carolina leper, who is now in Washington, D. C., Secretary Cortelyou has recently issued a statement in which he asserts that while he has authority to make regulations to prevent the introduction of contagious or infectious diseases into a state or territory or the District of Columbia, he has no authority to make a rule whereby this leper can be deported from Washington, or whereby North Carolina can be required to receive him.

---

The directors of the North Carolina State Hospital, Morganton, at a recent meeting, entered a formal protest against the recent action of the State Hospital Commission in declining to add to the existing plant of the State Hospital for the Insane. Morganton, until the State Hospital for the Insane at Raleigh had been enlarged and made to accommodate as many as the former institution; and furthermore, in ordering all tuberculosis cases in the Raleigh institution to be conveyed to the Morganton institution, which is situated in the mountains.

Two gold medals, which are given every three years by the International Conference on Tuberculosis for work done in advancing the fight against tuberculosis, have been awarded to Mr. Henry Phipps, of New York, and Dr. Frederick Althoff, of Berlin.

---

By a resolution of the Berlin Council it is directed that in the coming winter 8,000 poor school children shall receive warm dinners in the soup kitchens. The same privilege is to be extended to children not of school age on the recommendation of charitable societies.

---

The Supreme Court of Georgia had just decided that when an employer summons a physician to car efor an employee who has become suddenly ill, the employer is not liable for the bill unless there is a definite agreement between the employer and employee that the former shall be liable.

---

The United States Civil Service Commission announces an examination on January 13, 1909, at the regular locations throughout the United States, for a medical interne (female) in the Government Hospital for the Insane, Washington, at $600 a year, with maintenance.

---

The members of the Association of Military Surgeons of the United States were shown many social attentions while holding their seventeenth annual meeting in Atlanta.

On their first afternoon in the city, the visiting delegates were taken for an automobile ride, the next, as guests of the State Fair Association. They had all the privileges of the fair extended to them, scattering about the grounds at the Piedmont Park, and occupying boxes at the races.

The Fulton Medical Society gave a brilliant reception at the Piedmont Driving Club in honor of the visiting Surgeons to which two hundred and fifty guests were invited. The entire house was artistically decorated, and music in the ballroom added to the enjoyment of the occasion.

The guests were received by Dr. Willis Westmoreland, chairceiving party including Dr. and Mrs. Bates Block, Dr. and Mrs. E. C. Davis, Dr. and Mrs. Hugh Lokey, and Dr. and Mrs. Lindorm. A buffet supper was served at ten o'clock.

A barbecue at Morgan's 'cue grounds given by the Local Military Organization was the concluding social feature and was very much enjoyed.

---

At the annual meeting of the Sixth District Medical Association, held in Macon November 11, Dr. Alfred F. White, Flovilla, was elected president; Dr. James W. Cowart, Walden, vice-president, and Dr. Eugene B. Elder, Macon, secretary-treasurer (re-elected). The next meeting will be held at Indian Springs.

